# Sex differences and risk factors for postoperative complications following catheter ablation for pulmonary vein isolation in non-valvular atrial fibrillation: A retrospective cohort study

**DOI:** 10.1097/MD.0000000000042753

**Published:** 2025-06-13

**Authors:** Huasheng Lv, Fengyu Sun, Yongqiang Fan, Baopeng Tang, Yanmei Lu

**Affiliations:** a Department of Pacing and Electrophysiology, The First Affiliated Hospital of Xinjiang Medical University, Urumqi, China; b Department of Cardiac Electrophysiology and Remodeling, The First Affiliated Hospital of Xinjiang Medical University, Urumqi, China.

**Keywords:** atrial fibrillation, complications, pulmonary vein isolation, sex differences

## Abstract

Atrial fibrillation (AF) is the most common arrhythmia, significantly increasing the risk of adverse events such as stroke, heart failure, and cognitive impairment. catheter ablation is a first-line treatment for AF, with pulmonary vein isolation (PVI) as a common procedure. Although studies have reported sex-based differences in complication rates following PVI, these findings remain controversial. This study aimed to explore sex differences and identify independent risk factors associated with complications after PVI in non-valvular AF patients. This retrospective cohort study included 1092 patients with non-valvular AF who underwent PVI at the First Affiliated Hospital of Xinjiang Medical University between January 2018 and December 2021. The patients were divided into male and female groups, with propensity score matching used to reduce baseline differences. Data on clinical characteristics, intraoperative variables, and postoperative complications were collected. The primary outcome was the occurrence of complications after PVI, categorized into overall, mild, and major complications. Multivariate logistic regression analysis was performed to identify independent risk factors for complications. The study found that female patients experienced a higher incidence of postoperative complications compared to male patients (30.38% vs 19.89%, *P* = .001). The female group had significantly higher rates of pericardial effusion (20.17% vs 12.71%, *P* = .007) and mild complications, such as vagal hyperactivity (3.87% vs 1.38%, *P* = .036). Multivariate logistic regression revealed that female sex, obesity, New York Heart Association functional class ≥ II, and ablation of non-pulmonary veins were significantly associated with overall and mild complications. Sex differences significantly influence the occurrence of postoperative complications after PVI in non-valvular AF patients, with female patients at a higher risk. Targeted interventions considering these risk factors may improve patient outcomes. Further research is required to explore the underlying mechanisms driving these differences.

## 1. Introduction

Atrial fibrillation (AF) is the most common persistent arrhythmia, significantly increasing the risk of death, stroke, heart failure, cognitive impairment, and dementia, and seriously affecting the quality of life of patients.^[1]^ The prevalence of AF increases with age, and as the population ages, AF will place a significant burden on society and the healthcare system.^[2]^ Catheter ablation (CA) has become the first-line treatment for patients with AF.^[3]^ The main surgical methods include radiofrequency ablation (RFA), cryoballoon ablation (CBA), and pulsed field ablation (PFA), which are essential for achieving pulmonary vein isolation (PVI). The overall incidence of complications after PVI surgery during hospitalization ranges from 2.80% to 6.29%.^[4–6]^ With the development and widespread application of ablation technology, the incidence of complications is decreasing, and the postoperative complications of PVI cannot be ignored. Early studies have shown that the incidence of postoperative complications in female patients is higher than that in male patients, and female sex is an independent risk factor for postoperative complications.^[5,7–9]^ The impact of sex on the perioperative complications of PVI in patients with non-valvular AF is still controversial. Therefore, this study aimed to clarify the correlation between sex and postoperative complications of PVI, analyze the independent risk factors that affect the occurrence of postoperative complications in patients with non-valvular AF, and enable AF patients to benefit from personalized prevention and treatment, providing theoretical clinical guidance for patients to safely survive the perioperative period.

## 2. Methods

### 2.1. Research object

Patients with AF who underwent PVI at the Heart Center of the First Affiliated Hospital of Xinjiang Medical University between January 2018 and December 2021 were retrospectively selected. Inclusion criteria: patients with non-valvular AF who underwent PVI for the first time and were ≥ 18 years old; all selected patients met the class I or class II indications of CA for AF.^[3]^ All the selected patients signed an informed consent form for surgery. Exclusion criteria were as follows: age < 18 years; patients with valvular AF or with severe mitral and tricuspid valve lesions who did not undergo surgical treatment; thrombus in the left atrium or left atrial appendage; and PVI ablation ≥ 2 times. In this study, 1468 consecutive patients with non-valvular AF who underwent PVI were included. After screening according to the inclusion and exclusion criteria, 1092 patients were finally included, which were divided into a male group (696 cases) and a female group (396 cases). The study found that sex differences in postoperative complications of PVI may be closely related to BMI, age of onset, and comorbidity during ablation.^[4,10–14]^ In order to reduce the deviation of general data between male and female groups, In this study, 696 male patients and 396 female patients were analyzed with 1:1 propensity score matching (PSM), with grouping variables as dependent variables and age, BMI and complications during ablation as covariates. Finally, 362 pairs of patients were matched and included in the study.

This study has been approved by the Ethics Committee of the First Affiliated Hospital of Xinjiang Medical University, and the approval number is 231124-05. Given the retrospective nature of the study, informed consent was waived by the Ethics Committee as it involved the use of anonymized patient data that did not pose additional risk to participants.

### 2.2. Collection of clinical data

The patients’ sex, age, nationality, AF type, BMI, New York Heart Association (NYHA), CHA2DS2-VASc score, complications during ablation heart failure, hypertension, coronary heart disease, diabetes, embolism history, hypertrophic cardiomyopathy (HCM), obstructive sleep apnea-hypopnea syndrome, and previous cardiac surgery history (after heart valve surgery, after heart bypass surgery, with pacemaker and coronary artery). Laboratory examination indicators: PT, international normalized ratio, AST, ALT, albumin, and creatinine levels; improve TTE to evaluate patients’ cardiac function and valve condition and record left ventricular ejection fraction (LVEF), left atrial diameter, and right atrial diameter. Improve transesophageal echocardiography to determine whether there is thrombosis in the left atrium and left atrial appendage, exclude absolute contraindications for surgery, Improve the CT angiography of the left atrial pulmonary vein, and clarify the pulmonary vein structure.

Operation-related indicators: Record the duration of PVI operation, operation methods, presence of intraoperative LAAO, presence of electrical cardioversion during operation, presence of other arrhythmia ablation, presence of wireless ablation, and presence of ablation outside the trigger part of the pulmonary vein.

### 2.3. Definition and classification of complications

In this study, the common in-hospital complications of PVI were determined by using the diagnosis and program codes in ICD-9-CM and ICD-10-CM, which were the same as those described in previous published studies. ^[7,8,15–17]^ Complications were defined as the occurrence of operation-related safety complex events during or after PVI, and the main outcome was that at least 1 complication occurred after PVI during hospitalization. Because patients may have more than 1 complication, we counted the patients with at least 1 complication and counted them as overall complications. According to the severity of complications, it is divided into mild complications and major complications; Major complications are defined as those that cause serious injury or death, require reoperation and other intervention treatments, and prolong the hospital stay for more than 48 hours, including cardiac tamponade, bleeding requiring blood transfusion, severe pulmonary vein stenosis, severe esophageal injury (esophageal ulcer or atrial-esophageal fistula), postoperative embolic events (acute cerebral infarction or pulmonary embolism) and acute postoperative death. Mild complications included a small amount of pericardial effusion, vascular complications (puncture site bleeding, hematoma, pseudoaneurysm), mild esophageal injury, hyperactivity of the vagus nerve, pulmonary complications (lung infection, pleural effusion, post-injury syndrome), and phrenic nerve paralysis.

### 2.4. Statement

The production of this submitted work did not involve the use of artificial intelligence-assisted technologies such as Large Language Models, chatbots, or image creators.

### 2.5. Statistical methods

The data in this study were analyzed using R4.0.0, the statistical description of the measurement data with normal distribution and homogeneous variance was expressed by the mean standard deviation, and 2 independent samples were used for the comparison between groups. If the measurement data were not normally distributed, the median (interquartile interval) was used, and the Mann-Whitney rank sum test was used for comparison between groups. Counting data were expressed as percentages (%), and the Pearson χ² test was used. Use R4.0.0 to carry out 1:1 PSM analysis, calculate the tendency score, with a caliper value of 0.1, and match according to the nearest neighbor matching method. The ratio of complications between men and women was counted, and the relationship between sex and overall complications, mild complications, and major complications after PVI in the 2 groups was analyzed using SPSS 26.0 (Chicago) for single factor and multivariate conditional logistic regression. Take α = 0.05 as the statistical test level.

## 3. Results

### 3.1. Comparison of clinical and surgical data between the 2 groups

After matching, there were significant differences in the clinical baseline data of the 2 groups (*P* < .001) in the CHA_2_DS_2_-VASc score, aspartate aminotransferase (AST), and non-PV triggered ablation, and there were no differences in other indexes (Table 1).

**Table 1:** Comparison of baseline data, auxiliary examination and surgical data after matching.

Variables		After matching		
		Male (n = 362)	female (n = 362)	*P*–value
Age (yr)		64.00 (57.00, 71.00)	65.00 (56.00, 72.00)	.997
Ethnicity (n, %)				
Han nationality		282 (77.90)	302 (83.43)	.060
Ethnic minorities		80 (22.10)	60 (16.57)	
AF type (n, %)				
Paroxysmal AF		220 (60.77)	231 (63.81)	.399
Persistent AF		142 (39.23)	131 (36.19)	
BMI (kg/m^2^)		26.00 (23.00, 28.00)	25.00 (23.00, 28.00)	.352
NYHA (n, %)				
Grade I		61 (16.85)	60 (16.57)	.975
Grade II/III/IV		48 (13.26)	50 (13.81)	
CHA_2_DS_2_-VASc points		2.00 (1.00, 2.00)	3.00 (2.00, 4.00)	<.001
Complication (n, %)	HF	54 (14.92)	54 (14.92)	1.000
	Coronary heart disease	112 (30.94)	111 (30.66)	.936
	Hypertension	191 (52.76)	196 (54.14)	.709
	Diabetes	58 (16.02)	60 (16.57)	.841
	History of embolism	58 (16.02)	53 (14.64)	.606
	HCM	10 (2.76)	12 (3.31)	.665
	OSAHS	42 (11.60)	38 (10.50)	.635
	History of cardiac surgery	79 (21.82)	77 (21.27)	.857
Combined with other arrhythmia		200 (55.25)	198 (54.70)	.881
Length of stay (d)		6.00 (5.00, 7.00)	6.00 (5.00, 8.00)	.221
Duration of disease (mo)		20.00 (12.00, 27.00)	20.00 (12.00, 27.00)	.590
PT (s)		12.80 (11.70, 15.00)	12.70 (11.70, 15.00)	.634
International standardized ratio		1.12 (1.02, 1.34)	1.11 (1.02, 1.38)	.835
Albumin (g/L)		41.40 (38.80, 44.45)	41.30 (38.62, 44.50)	.816
AST (U/L)		22.95 (17.50, 28.07)	20.00 (15.93, 27.00)	.004
ALT (U/L)		22.52 (16.13, 32.93)	21.25 (14.70, 33.69)	.155
Creatinine (µmol/L)		74.00 (62.41, 85.00)	72.50 (61.00, 84.45)	.515
LVEF (mm)		60.12 (58.00, 64.00)	62.00 (58.00, 65.00)	.159
LAD (mm)		39.00 (37.00, 42.00)	39.00 (37.00, 42.00)	.340
RAD (mm)		37.00 (35.00, 39.00)	37.00 (35.00, 39.00)	.066
Surgical duration (min)		130.00 (120, 130)	130.00 (120, 130)	.920
Surgical method (n, %)				
RFA		314 (86.74)	312 (86.19)	.975
CBA		48 (13.26)	50 (13.81)	
Intraoperative LAAO (n, %)		52 (14.36)	62 (17.12)	.308
Intraoperative electroconversion (n, %)		132 (36.46)	138 (38.12)	.645
Ablation of other arrhythmias (n, %)		90 (24.86)	104 (28.73)	.240
Linear ablation (n, %)		142 (39.23)	141 (38.95)	.939
Non-PV triggered ablation (n, %)		98 (27.07)	162 (44.75)	<.001

AF = atrial fibrillation, ALT = alanine aminotransferase, AST = aspartate transferase, BMI = body mass index, CBA = cryoballoon ablation, HCM = hypertrophic cardiomyopathy, HF = heart failure, LAAO = left atrial appendage occlusion, NYHA = New York Heart Association, OSAHS = obstructive sleep apnea-hypopnea syndrome, PT = prothrombin time, PV = pulmonary vein, RFA = radiofrequency ablation.

### 3.2. Composition ratio of complications

For the postoperative complications of PVI, there were significant statistical differences between the 2 groups in overall complications, mild complications, pericardial effusion, micro and small amounts of pericardial effusion, and complications of vagal hyperfunction. There was no statistical difference in major complications between the 2 groups; however, the distribution ratios were different. The female group had more complications of cardiac tamponade and severe esophageal injury than the male group, and there were no acute deaths in either group (Table 2).

**Table 2:** Comparison of postoperative complications between the 2 groups.

All complications (n, %)	After matching			
	Male (n = 362)	Female (n = 362)	χ^2^	*P*-value
Overall complication	72 (19.89)	110 (30.38)	10.598	.001
Pericardial effusion	46 (12.71)	73 (20.17)	7.331	.007
Vascular complications	28 (7.73)	33 (9.12)	0.448	.504
Esophageal injury	22 (6.08)	28 (7.73)	0.773	.379
Mild complications	60 (16.57)	100 (27.62)	12.837	<.001
Micro/small amount of pericardial effusion	32 (8.84)	52 (14.36)	5.387	.020
Mild vascular complications	24 (6.63)	31 (8.56)	0.964	.326
Mild esophageal injury	17 (4.70)	22 (6.08)	0.678	.410
Pulmonary complications	8 (2.21)	16 (4.42)	2.758	.097
Hyperactivity of vagus nerve	5 (1.38)	14 (3.87)	4.378	.036
Phrenic nerve paralysis	2 (0.55)	4 (1.10)	0.672	.412
Major complications	30 (8.29)	33 (9.12)	0.156	.692
Cardiac tamponade	8 (2.21)	17 (4.70)	3.356	.067
Suffer a massive hemorrhage	4 (1.10)	2 (0.55)	0.672	.412
Severe pulmonary vein stenosis	1 (0.28)	1 (0.28)	0.000	1.000
Severe esophageal injury	5 (1.38)	6 (1.66)	0.092	.761
Postoperative embolic events	14 (3.87)	8 (2.21)	1.688	.194
Acute death	0	0	0.000	1.000

### 3.3. Univariate logistic regression analysis of complications

**Figure 1.**
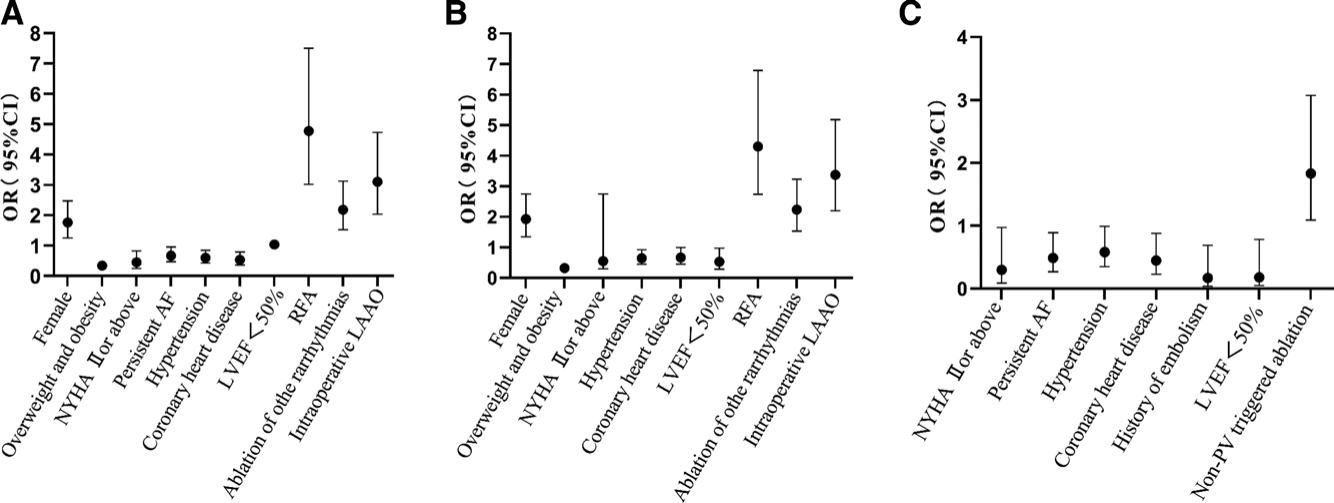
Univariable logistic regression. (A) Overall complications. (B) Mild complications. (C) Major complications.

Variables such as female sex, age, overweight and obesity, NYHA functional class Ⅱ or above, CHA_2_DS_2_-VASc ≥ 2 points, persistent AF, hypertension, coronary artery disease, history of previous embolism, LVEF < 50%, left atrial diameter augmentation, RFA, ablation of other arrhythmias, intraoperative LAAO, linear ablation, and non-PV triggered ablation were substituted into the logistic regression equation for univariate analysis. It showed that female, overweight, and obesity, NYHA functional class Ⅱ or above, persistent AF, hypertension, coronary artery disease, LVEF < 50%, RFA, ablation of other arrhythmias, and intraoperative LAAO were associated with overall complications after PVI (*P* < .05) (Table 3, Fig. 1A); female sex, overweight and obesity, NYHA functional classⅡ or above, hypertension, coronary artery disease, LVEF < 50%, RFA, intraoperative LAAO, LVEF < 50%, RFA, ablation of other arrhythmias, and intraoperative LAAO were associated with mild complications after PVI (*P* < .05) (Table 3, Fig. 1B); NYHA functional class Ⅱ or above, persistent AF, hypertension, coronary artery disease, history of previous embolism, LVEF < 50%, and intraoperative LAAO were associated with major complications after PVI (*P* < .05) (Table 3, Fig. 1C).

**Table 3:** Univariate logistic regression analysis.

Index		Overall complication		Mild complications		Major complications	
		OR (95% CI)	*P*-value	OR (95% CI)	*P*-value	OR (95% CI)	*P*-value
Female		1.76 (1.25–2.47)	.001	1.92 (1.34–2.75)	<.001	1.11 (0.66–1.86)	.693
Age (yr)	<65	Reference		Reference		Reference	
	65–74	0.97 (0.60–1.57)	.904	0.76 (0.47–1.24)	.275	1.40 (0.60–3.28)	.442
	≥75	1.22 (0.73–2.01)	.449	1.05 (0.63–1.74)	.863	2.05 (0.86–4.85)	.104
Overweight and obesity		0.34 (0.24–0.48)	.001	0.32 (0.22–0.46)	<.001	0.60 (0.35–1.03)	.065
NYHA functional class Ⅱ or above		0.45 (0.25–0.82)	.009	0.55 (0.30–2.75)	.048	0.30 (0.09–0.97)	.044
CHA_2_DS_2_-VASc ≥ 2 points		0.84 (0.60–1.20)	.353	1.04 (0.91–1.19)	.566	0.91 (0.53–1.56)	.730
Persistent AF		0.67 (0.47–0.95)	.026	0.80 (0.55–1.16)	.242	0.49 (0.27–0.89)	.019
Hypertension		0.60 (0.43–0.84)	.003	0.65 (0.45–0.92)	.016	0.58 (0.35–0.99)	.044
Coronary heart disease		0.53 (0.36–0.79)	.002	0.67 (0.45–0.99)	.047	0.45 (0.23–0.88)	.019
History of embolism		0.75 (0.46–1.22)	.245	0.97 (0.57–1.58)	.895	0.17 (0.04–0.69)	.013
LVEF<50%		1.03 (1.00–1.05)	.024	0.53 (0.29–0.97)	.038	0.18 (0.05–0.78)	.022
LAD enlargement		0.98 (0.95–1.03)	.454	0.97 (0.93–1.01)	.148	0.79 (0.44–1.36)	.380
RFA		4.77 (3.02–7.50)	<.001	4.30 (2.73–6.79)	<.001	1.71 (0.87–3.34)	.117
Ablation of other arrhythmias		2.18 (1.52–3.12)	<.001	2.23 (1.53–3.23)	<.001	1.10 (0.62–1.96)	.739
Intraoperative LAAO		3.10 (2.03–4.73)	<.001	3.37 (2.20–5.18)	<.001	0.57 (0.24–1.36)	.204
Linear ablation		0.88 (0.62–1.24)	.467	0.89 (0.62–1.27)	.516	1.03 (0.61–1.74)	.919
Non-PV triggered ablation		1.27 (0.90–1.80)	.173	1.09 (0.76–1.57)	.635	1.83 (1.09–3.07)	.023

AF = atrial fibrillation, LAAO = left atrial appendage occlusion, LAD = left atrial diameter, LVEF = left ventricular ejection fraction, NYHA = New York Heart Association, PV = pulmonary vein, RFA = radiofrequency ablation.

### 3.4. Multivariate logistic regression analysis of complications

**Figure 2.**
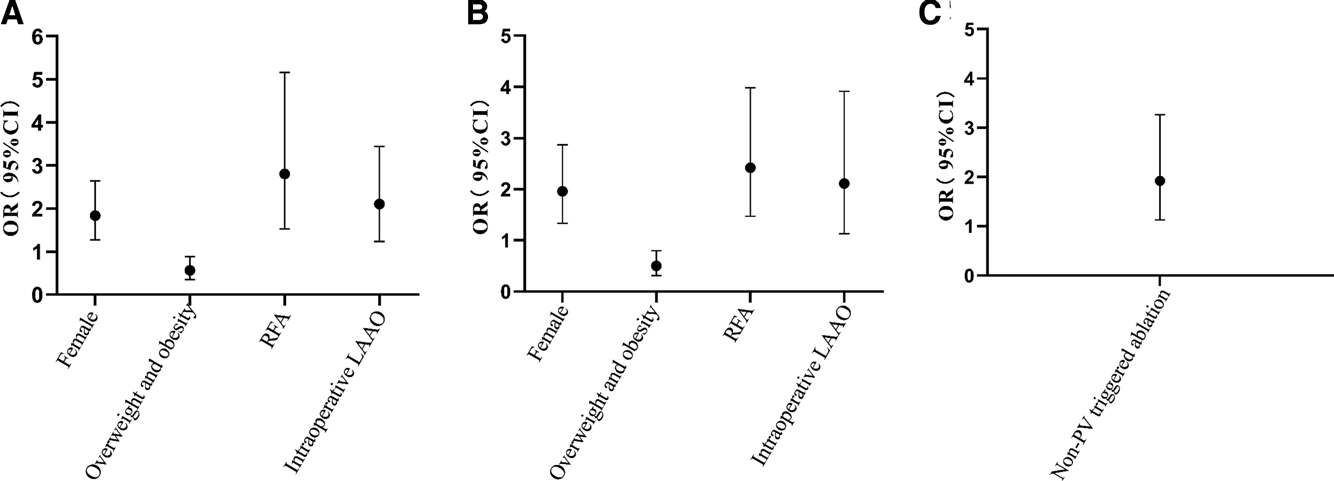
Multivariable logistic regression. (A) Overall complications. (B) Mild complications. (C) Major complications.

Multivariate logistic regression analysis included statistically significant indicators in the univariate logistic regression analysis. Differences in female sex, overweight and obesity, RFA, and intraoperative left atrial appendage closure were significantly associated with overall complications and mild complications (Table 4, Fig. 2A and B). Non-PV triggered ablation was associated with major complications (Table 4, Fig. 2C).

**Table 4:** Multivariate logistic regression analysis.

Index	Overall complication		Mild complications		Major complications	
	OR (95% CI)	*P*-value	OR (95% CI)	*P*-value	OR (95% CI)	*P*-value
Female	1.83 (1.27–2.64)	.001	1.96 (1.33–2.87)	.001		
Overweight and obesity	0.56 (0.35–0.88)	.013	0.50 (0.31–0.80)	.004		
NYHA functional class Ⅱ or above	0.62 (0.19–2.05)	.436	0.92 (0.27–3.18)	.896	2.35 (0.38–14.66)	.360
Persistent AF	1.18 (0.73–1.90)	.501			0.71 (0.35–1.46)	.355
Hypertension	0.72 (0.47–1.11)	.141	0.72 (0.46–1.12)	.146	0.81 (0.44–1.48)	.489
Coronary heart disease	0.59 (0.34–1.03)	.062	0.85 (0.50–1.47)	.568	0.84 (0.37–1.90)	.681
History of embolism					0.28 (0.06–1.34)	.111
LVEF<50%	0.95 (0.30–3.08)	.938	0.66 (0.19–2.30)	.513	0.27 (0.03–2.29)	.229
RFA	2.80 (1.52–5.16)	.001	2.11 (1.13–3.91)	.018		
Ablation of other arrhythmias	1.11 (0.68–1.80)	.686	1.11 (0.67–1.85)	.680		
Intraoperative LAAO	2.10 (1.23–3.44)	.003	2.42 (1.47–3.98)	.001		
Non-PV triggered ablation					1.92 (1.13–3.26)	0.016

AF = atrial fibrillation, LAAO = left atrial appendage occlusion, LVEF = left ventricular ejection fraction, NYHA = New York Heart Association, PV = pulmonary vein, RFA = radiofrequency ablation.

### 3.5. Subgroup analysis of related interventions after atrial fibrillation ablation

This study conducted subgroup analyses to investigate the impact of LAAO and linear ablation on complications following AF ablation, as well as the relationships among significant indicators in multivariate logistic regression. Patients were divided into the following groups: the PVI-only group (n = 344), the LAAO group (n = 97), the linear-Abl group (n = 271), representing patients who underwent linear ablation, and the both group (n = 12), which included patients who received both left atrial appendage occlusion and linear ablation (Table 5). Significant intergroup differences were observed in complication rates. The overall complication rate in the LAAO group was 46.39%, significantly higher than that in the Linear-Abl group (22.88%, *P* < .001). The rate of mild complications in the LAAO group was 42.27%, significantly higher than that in the Linear-Abl group (19.56%, *P* < .001). For major complications, the rate in the LAAO group was 5.15%, compared to 8.86% in the Linear-Abl group, but this difference was not statistically significant (*P* = .595). Analysis of the distribution of key factors among the groups revealed no statistically significant differences in gender, overweight and obesity, persistent AF, coronary heart disease, hypertension, ablation of other arrhythmias, non-PV triggered ablation, or RFA (radiofrequency ablation) (*P* > .05). However, the distributions of NYHA functional class II or higher and LVEF < 50% approached statistical significance (*P* = .055 and *P* = .053, respectively).

**Table 5:** Subgroup analysis of related interventions after atrial fibrillation ablation.

Variables (n, %)	PVI-only (n = 344)	LAAO (n = 97)	Linear-Abl (n = 271)	Both (n = 12)	Statistic	*P*
Overall complication	70 (20.35)	45 (46.39)	62 (22.88)	5 (41.67)	29.95	<.001
Pericardial effusion	48 (13.95)	30 (30.93)	38 (14.02)	3 (25.00)	18.17	<.001
Vascular complications	10 (2.91)	39 (40.21)	9 (3.32)	3 (25.00)	153.98	<.001
Esophageal injury	21 (6.10)	10 (10.31)	18 (6.64)	1 (8.33)	2.16	.540
Mild complications	60 (17.44)	41 (42.27)	53 (19.56)	6 (50.00)	33.70	<.001
Micro or small amount of pericardial effusion	30 (8.72)	27 (27.84)	25 (9.23)	2 (16.67)	29.50	<.001
Mild vascular complications	5 (1.45)	39 (40.21)	8 (2.95)	3 (25.00)	178.94	<.001
Mild esophageal injury	18 (5.23)	5 (5.15)	15 (5.54)	1 (8.33)	0.24	.970
Pulmonary complications	8 (2.33)	0 (0.00)	13 (4.80)	3 (25.00)	–	<.001
Hyperactivity of vagus nerve	7 (2.03)	5 (5.15)	6 (2.21)	1 (8.33)	–	.132
Phrenic nerve paralysis	2 (0.58)	2 (2.06)	1 (0.37)	1 (8.33)	–	.033
Major complications	33 (9.59)	5 (5.15)	24 (8.86)	1 (8.33)	1.89	.595
Cardiac tamponade	11 (3.20)	0 (0.00)	13 (4.80)	1 (8.33)	–	.054
Suffer a massive haemorrhage	5 (1.45)	0 (0.00)	1 (0.37)	0 (0.00)	–	.420
Severe pulmonary vein stenosis	1 (0.29)	0 (0.00)	1 (0.37)	0 (0.00)	–	1.000
Severe esophageal injury	3 (0.87)	5 (5.15)	3 (1.11)	0 (0.00)	–	.050
Postoperative embolic events	15 (4.36)	0 (0.00)	7 (2.58)	0 (0.00)	–	.143
Female	179 (52.03)	42 (43.30)	136 (50.37)	4 (33.33)	3.66	.301
Overweight and obesity	251 (72.97)	67 (69.07)	193 (71.48)	9 (75.00)	0.66	.883
NYHA functional class Ⅱ or above	58 (16.86)	9 (9.28)	31 (11.48)	0 (0.00)	7.59	.055
Persistent AF	136 (39.53)	37 (38.14)	93 (34.44)	7 (58.33)	3.89	.273
Coronary heart disease	109 (31.69)	29 (29.90)	80 (29.63)	5 (41.67)	1.00	.801
Hypertension	181 (52.6 )	54 (55.67)	145 (53.70)	7 (58.33)	0.41	.938
LVEF<50%	59 (17.15)	9 (9.28)	32 (11.85)	0 (0.00)	7.68	.053
Ablation of other arrhythmias	89 (25.87)	32 (32.99)	71 (26.30)	2 (16.67)	2.71	.439
Non-PV triggered ablation	114 (33.14)	43 (44.33)	97 (35.93)	6 (50.00)	5.17	.160
RFA	303 (88.08)	80 (82.47)	236 (87.41)	12 (100.00)	3.97	.265

AF = atrial fibrillation, LAAO = left atrial appendage occlusion, LVEF = left ventricular ejection fraction, NYHA = New York Heart Association, PVI = pulmonary vein isolation, RFA = radiofrequency ablation.

## 4. Discussion

### 4.1. Relationship between sex and complications

Evidence shows that there are sex differences in patients with AF. Female patients have more serious symptoms, lower quality of life scores, and higher ineffective rates of antiarrhythmic drugs.^[18]^ The proportion of women receiving ablation treatment is still lower than that of men.^[4,18–22]^ The reasons for sex differences are mainly related to delayed ablation time, greater atrial fibrosis, more complex clinical features, and higher incidence of non-pulmonary vein trigger sites in women.^[23]^ Socioeconomic factors also influence women’s decisions to make PVI.^[24]^ The improvement in the quality of life of male and female patients after PVI is similar;however, at all time points, the overall incidence of complications reported by female patients is higher than that of male patients.^[7,8,11]^ It has been suggested that differences in the size, shape, and atrial substrate of the female left atrium may increase the risk of perforation during atrial septal puncture and catheter operation^[10,25]^ and extensive endocardial injury.^[26]^ After PSM and multivariate logistic regression analysis, female sex was still an independent risk factor for overall complications after PVI. The increase in the incidence of complications in women cannot be explained only by age and comorbidity. Female-specific biological factors may play a role in the increased the risk of complications.

Sex differences in postoperative complications of PVI are still controversial in the CABANA trial.^[10]^ It is found that women are older and had more obvious symptoms than men, but no sex difference was observed in the primary and secondary clinical outcomes. The results of the CABANA trial support that CA is an effective treatment strategy for both women and men.

Lack of consideration of gender specificity and sex-specific pathophysiology in medical research.^[27]^ Identifying and reducing gender bias in medical decision-making and result reporting may promote fairer medical services, and bringing gender dimensions into biomedical exploration is also an important aspect of optimizing AF patient care. The 2020 ESC AF management guidelines emphasize that women should undergo equal treatment to prevent AF-related complications, and women with paroxysmal or persistent AF should receive ablation surgery in time.^[3]^

### 4.2. Relationship between ablation methods and complications

At present, the surgical procedures for PVI include RFA, CBA, and PFA, and the incidence of different ablation procedures varies. The incidence of cardiac tamponade and esophageal injury after CBA + PVI is significantly lower than that after RFA + PVI.^[28,29]^ CBA is associated with phrenic nerve paralysis.^[28]^ Compared with RFA, CBA has a lower risk of pulmonary vein stenosis due to tissue contraction because the basic tissue structure is preserved after ablation.^[30,31]^ PFA is a new type of energy ablation that releases a pulsed electric field and produces electroporation on the cell membrane, leading to cell death and increased safety.^[32–34]^ It still needs a large sample and prospective study for further discussion. RFA and CBA ablation are still the most commonly used ablation methods in clinic. With the development of 3-dimensional mapping technology, supplemented by ultrasound guidance of puncture blood vessels, intracavity ultrasound in the operation center can image in real time and identify related complications at an early stage.

### 4.3. Relationship between intraoperative LAAO and complications

The left atrial appendage is the most important cardiac source of thromboembolism in patients with non-valvular AF. As a nondrug treatment to prevent thromboembolism in patients with non-valvular AF, occlusion of the LAA with an occluder has become an effective alternative to anticoagulant therapy.^[17]^ Compared with warfarin, LAAO is safe and effective in reducing the risk of stroke when long-term anticoagulation therapy is not ideal or contraindication.^[35,36]^ The incidence of hospitalization adverse events in women after LAAO is higher than that in men, Darden et al.^[17]^ From 2016 to 2019, 49,357 patients with LAAO were selected, with an overall complication rate of 4.9%. Women were more likely to have any adverse events (6.3% vs 3.9%, *P* < .001) or major adverse events (4.1% vs 2.0%; *P* < .001), especially pericardial effusion requiring drainage (1.2% vs 0.5%) or bleeding complications (1.7% vs 0.8%), and were more likely to die (0.3% vs 0.1%, *P* < .001). Watchman’s experience in Europe shows that women are the predictors of pericardial effusion and cardiac tamponade on the 30th day, although the overall low incident rate is 1%.^[37]^ The data from the PROTECT-AF and please trials did not show any sex differences in the incidence of complications.^[38,39]^ In this study, the number of men with LAAO before PSM was higher than that of women, there was no significant difference between them after matching, and no sex difference was found in the complications after PVI combined with LAAO. A small study of 78 people found that there were more women than men with left atrial appendage device-related thrombosis, but this was not statistically significant and was not a related factor in the multivariate analysis.^[40]^ Women are underrepresented in clinical trials and retrospective studies of AF, which makes it difficult to interpret the data and understand the real risks and benefits of different management and treatment strategies.

The subgroup analysis further confirmed that LAAO is associated with a higher incidence of complications. The overall complication rate and the rate of mild complications in the LAAO group were significantly higher than those in the Linear-Abl group. Although the difference in the rate of major complications between the groups did not reach statistical significance, the trend observed in the LAAO group suggests a potential risk. Further studies with larger sample sizes are needed to more thoroughly investigate the impact of LAAO on major complications.

PVI combined with LAAO is a suitable scheme for non-valvular AF patients with a stroke risk and poor anticoagulation treatment. Compared with PVI alone, PVI can save hospitalization and long-term anticoagulation treatment costs and reduce compliance with anticoagulation treatment. To reduce the risk of surgery, guide the selection of the appropriate size and shape of the left atrial appendage occluder, master the occluder skillfully, and continue to develop safer instruments;long-term follow-up is still needed to further verify the safety and effectiveness of the operation.

### 4.4. Relationship between non-PV vein-triggered ablation and complications

Extrapulmonary vein ablation based on PVI is developing rapidly to obtain better rhythm control in patients with persistent AF. Non-pulmonary vein-triggered ablation sites mainly include the left atrial posterior wall, superior vena cava, coronary sinus, marginal crest, Marshall vein, oval fossa, and left atrial appendage.^[3]^ Women have more non-pulmonary vein trigger lesions, and increasing ablation of non-pulmonary vein sites may increase the risk of major complications, such as cardiac perforation and cardiac tamponade.^[41–43]^ This study reached the same conclusion; however, most studies did not provide details about the specific ablation site and injury; therefore, it is difficult to assess the extent to which this may lead to an increase in the incidence of complications. At present, there is still no consensus on the best ablation strategy for AF patients, and surgeons should take measures according to each patient’s situation, existing evidence, and personal experience.^[27]^

### 4.5. Relationship between BMI and complications

This study found that being overweight or obese may be protective factors for overall and mild complications after PVI in patients with non-valvular AF. Obese patients often have larger blood vessels, which may be beneficial in reducing the risk of bleeding and blood vessel injury.^[44,45]^ In addition, studies have found that lower BMI is associated with an increased risk of cardiac tamponade. A recent Japanese study^[46]^ analyzed 59,789 AF patients (aged 65.6 ± 10.4 years) who received PVI treatment from 2016 to 2018. Multivariate analysis showed that BMI < 18.5kg/m^2^ was independently associated with an increased risk of cardiac tamponade (RR 1.42; 95% CI: 1.03–1.95) compared with patients with BMI in the range of 18.5 to 25 kg/m^2^, which may be due to the higher risk of bleeding in anticoagulant state, and the smaller left atrium in patients with low BMI, which may make it more difficult to manipulate the ablation catheter in the left atrium, resulting in mechanical damage to the left atrium wall. It is worth mentioning that weight management can improve lung function and fat distribution, and long-term sustained weight loss is related to significantly reducing AF load and maintaining sinus rhythm.^[47]^

### 4.6. Study limitations

This was a single-center retrospective study that balanced the differences in age, BMI, and complications during ablation between male and female groups using the PSM method. Compared with the study without PSM, the results are more reliable but may ignore the interaction between various indicators, which may increase the selection bias, leading to differences in some research results. The research conclusion needs to be further demonstrated by larger sample sizes and multicenter studies. There was no long-term follow-up in this study, and delayed complications could not be recorded, leading to certain limitations.

## 5. Conclusion

In summary, there are sex-related differences in complications between male and female patients. We could not fully clarify the reasons for the increased risk of adverse events in women. Further studies are needed to explore the mechanisms underlying sex differences. Thus, the overall safety of PVI is acceptable. We should pay attention to health education, early intervention on risk factors, close monitoring during and after operation, and clinicians’ mastery of PVI technology to improve the quality of life of patients with AF.

## Author contributions

**Conceptualization:** Huasheng Lv, Baopeng Tang.

**Data curation:** Huasheng Lv, Fengyu Sun, Yongqiang Fan.

**Funding acquisition:** Yanmei Lu.

**Writing – original draft:** Huasheng Lv.

**Writing – review & editing:** Huasheng Lv, Baopeng Tang, Yanmei Lu.
